# What Is the Role of Informal Healthcare Providers in Developing Countries? A Systematic Review

**DOI:** 10.1371/journal.pone.0054978

**Published:** 2013-02-06

**Authors:** May Sudhinaraset, Matthew Ingram, Heather Kinlaw Lofthouse, Dominic Montagu

**Affiliations:** 1 Global Health Sciences, University of California San Francisco, San Francisco, California, United States of America; 2 Metta Fund, Corte Madera, California, United States of America; 3 Absolute Return for Kids US, New York, New York, United States of America; Consejo Superior de Investigaciones Cientifics, Spain

## Abstract

Informal health care providers (IPs) comprise a significant component of health systems in developing nations. Yet little is known about the most basic characteristics of performance, cost, quality, utilization, and size of this sector. To address this gap we conducted a comprehensive literature review on the informal health care sector in developing countries. We searched for studies published since 2000 through electronic databases PubMed, Google Scholar, and relevant grey literature from The New York Academy of Medicine, The World Bank, The Center for Global Development, USAID, SHOPS (formerly PSP-One), The World Health Organization, DFID, Human Resources for Health Global Resource Center. In total, 334 articles were retrieved, and 122 met inclusion criteria and chosen for data abstraction. Results indicate that IPs make up a significant portion of the healthcare sector globally, with almost half of studies (48%) from Sub-Saharan Africa. Utilization estimates from 24 studies in the literature of IP for healthcare services ranged from 9% to 90% of all healthcare interactions, depending on the country, the disease in question, and methods of measurement. IPs operate in a variety of health areas, although baseline information on quality is notably incomplete and poor quality of care is generally assumed. There was a wide variation in how quality of care is measured. The review found that IPs reported inadequate drug provision, poor adherence to clinical national guidelines, and that there were gaps in knowledge and provider practice; however, studies also found that the formal sector also reported poor provider practices. Reasons for using IPs included convenience, affordability, and social and cultural effects. Recommendations from the literature amount to a call for more engagement with the IP sector. IPs are a large component of nearly all developing country health systems. Research and policies of engagement are needed.

## Introduction

In many developing nations, the informal sector provides the bulk of health care, particularly for the poor [1]. Literature on the subject of informal providers (IPs) resides in silos related to narrow fields and, to date, few researchers have evaluated the nature and impact of the informal provision of health care. In addition, while there is little evidence on the quality of care provided by IPs, there is general acceptance that IPs provide substandard care [2]. We assess the literature on the scope and practice, size and utilization patterns, quality, and reasons people use IPs. In addition, we summarize recommendations in regards to IPs in the developing world.

### Defining Informal Provider

While the precise definition of the term “informal health care providers” is inconsistent across studies [2] and we found no developed typology in the literature, we provide a working definition in order to identify relevant studies. We used a flexible set of criteria as opposed to specific delineations for characteristics such as duration of training or group membership. Such rigid definitions are inappropriately constrictive in light of significant contextual variation. To be classified as such, IPs must meet our first criteria below, and at least two of the remaining three criterion.

The set of definitional criteria include:

1. *Training:* IPs include those who have not received formally recognized training with a defined curriculum from an institution (i.e. government, NGO, or academic institution). IPs, however, typically have some level of informal training through apprenticeships, seminars, and workshops, and are typically not mandated by any formal institution.
2. *Payment*: IPs collect payment from patients served, not from institutions. One notable exception to this criterion involves NGO or other sponsored voucher programs, where informal providers exchange services or goods for payment from a sponsoring body in the form of reimbursement vouchers. Payment is usually, but not always, un-documented and tendered in cash. IPs are chiefly entrepreneurs.
3. *Registration and regulation:* IPs are not typically registered with any government regulatory body and operate outside of the purview of regulation, registration, or oversight by the government or other institutions
4. *Professional affiliation*: IP professional associations, if they exist, are primarily focused on networking and business activities and conduct minimal self-regulation.

IPs are a heterogeneous group of providers with differences in type of training, regulatory frameworks, and services provided; however, in this paper, we purposefully decided to take an inclusive approach and include in the results and discussions all types of informal providers in to one category for a number of reasons. First, local governments tend to think of providers outside the formal sector as one group with little knowledge on the size and utilization of this sector. A comprehensive global summary is needed given the lack of information known. Second, the current literature typically does not specify what type of informal provider is included or else includes multiple types of informal providers in the study. It is quite common that informal providers in different contexts are performing similar preventative and curative activities, even if they have different titles depending on their locale. Therefore, key lessons learned can translate to a variety of providers who are untrained and work outside regulatory frameworks. Because caregivers do not receive payment for services, and therefore fail to meet the first of our key definitional criteria, we excluded them even though they feature many of the same traits and practice in a similar fashion to other IPs. Community health workers who were trained by NGOs or governments are not included in the study. Only studies that specify “untrained” community health workers are included the study results.

## Methods

Using an expanded keyword search list, we searched electronic databases and websites for relevant published and grey literature. A larger study conducted in mid- 2010 on informal providers looked at multiple databases and languages and found widespread duplication of materials and language-based confusion of terms used to describe informal providers. On the basis of that experience we limited this search to only English language studies, and only two electronic datasets; supplemented with multi-language grey literature searches. We queried the following electronic databases for relevant published literature: PubMed, Google Scholar; and searched the following websites for relevant grey literature: The New York Academy of Medicine, The World Bank, The Center for Global Development, USAID, SHOPS (formerly PSP-One), The World Health Organization, DFID, Human Resources for Health Global Resource Center.

To identify studies conducted on IPs, a set of search terms was used (see Table 1). Inclusion criteria for the review were: studies conducted in a low or middle-income country (LMIC), conducted in 2000 or after, and reported data on a health outcome. We restricted inclusion only to articles where informal providers were the focus of the article, meaning that the search terms appeared in the title or abstract. The search identified approximately 3,000 articles, which were then screened to determine if the study occurred in a low- or middle-income country as defined by the World Bank. In total, 334 articles were included for review, of which 103 were over a decade old (pre-year 2000), and were therefore excluded from further analysis. Of the 231 remaining articles, 122 were chosen for data abstraction after reviewers ensured the studies met the inclusion criteria. The protocol for identifying studies can be found at: http://cl.ly/3y232d0k1E2R2c301R0k. This includes criteria for considering types of studies for review, electronic searches, and data extraction and management. Only studies based on primary data were included.

**Table 1:** Search Terms for Informal Providers.

Alternative healer	Less than fully qualified practitioner
Alternative health practitioner	Local medical practitioner
Alternative health provider	Medical detailer
Alternative medical practitioner	Non-graduate medical practitioner
Alternative medical provider	Non-registered health care provider
Alternative practitioner	Non-state actor
Alternative provider	Patent medicine vendor
Ayurved	Pharmacy worker
Ayurveda	Private sector
Community health worker	Quack
Compounder	Rural medical practitioner
Detailer	Rural practitioner
Drug seller	Semi-qualified provider
Drug vendor	Shopkeeper
Folk medicine	Traditional birth attendant
Folk practitioner	Traditional healer
Hakeem	Traditional medical practitioner
Healer	Traditional practitioner
Herbalist	Traditional provider
Homeopath	Traditional therapists
Indigenous practitioner	Unqualified allopathic provider
Individual practitioner	Unqualified provider
Informal provider	Untrained practitioner
Informal sector	Untrained provider
Lady health worker	Village doctor

Two researchers abstracted the data for the review. Each read and categorized all references and collected the following information for each: first author, year, title, region, country, type of IPs included, quality data, cost data, utilization data, size data, recommendations (two maximum per reference), type of study, type of intervention (if appropriate), intervention successful (if appropriate), disease discussed, and any important notes (i.e. target populations, % received care from IP vs. other provider; % received care from type of IP etc.). Data were entered into and analyzed using a Microsoft Excel database.

Due to a limited body of research on informal providers, the review includes all existing relevant research and types of study designs. There are inherent biases to including a range of study designs, including limitations in cross-sectional studies and the inability to establish causality when assessing interventions. In addition, publication bias may result in intervention studies due to the greater likelihood of publishing interventions with statistically significant results on specified outcomes. Size and utilization data are presented in Table 2. Study designs, sample size, and predictors of quality are reported in Table 3 for studies assessing the quality of IPs.

**Table 2:** Size and Utilization of the Informal Healthcare Sector.

Country	Study	IP Type	Utilization (% of healthcare provided by IP)	Size (% of providers that are informal)
*Bangladesh*				
	(Ahmed 2005)	Multiple	0.65	–
	(Ahmed, Hossain et al. 2009)	Multiple	–	0.88
	(Bhuiya and Book 2009)	Multiple	–	0.96
	(Hosain, Ganguly et al. 2005)	Multiple	0.77	–
	(Cockcroft, Milne et al. 2004)	Multiple	0.6	–
	(Levin, Rahman et al. 2001)	Multiple	0.65	–
*India*				
	(De Costa and Diwan 2007)	Untrained Provider, TBA	–	0.55
	(Kanjilal, Mondal et al. 2007)	RMPs	54%/19%	–
	(Rao 2005)	RMPs, Unknown	–	0.51
*Kenya*				
	(Amin, Marsh et al. 2003)	Multiple	0.33	–
	(Hamel, Odhacha et al. 2001)	CHW, Traditional Practitioner, Drug Sellers	9%/32%	–
*Laos*				
	(Sydara, Gneunphonsavath et al. 2005)			
*Mozambique*				
	(Gloyd, Floriano et al. 2001)	TBA	0.43	–
*Multiple*				
	(Brieger, Osamor et al. 2004)	Drug Sellers	15–82%, Median = 50%	–
	(WHO and Organization) 2002)	Traditional Medicine	60–90%	–
	(Greer, Akinpelumi et al. 2004)	Multiple	15–73%	–
	(Tawfik, Northrup et al. 2002)	Multiple	14–60%	–
*Nepal*				
	(Shankar, Partha et al. 2002)	Compounder	0.36	–
*Nigeria*				
	(Brieger, Salako et al. 2001)	Drug Sellers	0.36	–
	(Enato and Okhamafe 2006)	Drug Sellers	0.44	–
	(Salako, Brieger et al. 2001)	Drug Sellers	0.49	–
	(Oladepo, Salami et al. 2008)	Drug Sellers	0.39	–
*Tanzania*				
	(Battersby, Goodman et al. 2003)			
	(Corno 2008)	Multiple	0.13	–
*Thailand*				
	(Bryant and Prohmmo 2001)	Drug Sellers	55–77%	–
*Uganda*				
	(Jacobs, Whitworth et al. 2004)	Drug Sellers	0.35	–
	(Konde-Lule, Nakacubo Gitta et al. 2010)	Multiple	0.11	0.77
	(Twebaze 2001)	Drug Sellers/Traditional Healers	40/62%	–

**Table 3:** Studies reporting on quality of informal providers.

Author	Year	Sample	Study Design	Quality Predictors	Outcome	Significance
Abuya [3]	2009	n = 270 drug sellers in intervention; n = 288 control	RCT Intervention 10 administrative divisions	Adequacy of selling medicines; advice offered during client survey; knowledge	Malaria	+
Adu-Sarkodie [5]	2000	n = 50 pharmacy outlets received training; n = 50 control outlets with no training	RCT Intervention for training	Adequacy of drug provision	Urethral discharge	+
Ahmed [58]	2007	n = 445 Drug store salespeople; n = 509 village doctors; n = 490 community health workers	Cross-sectional convenience sample in rural Bangladesh of qualified vs. semi-qualified	Provider knowledge; management of diseases	Multiple outcomes	Overall, semi-trained providers scored higher on knowledge of risk factors and management of disease
Ahmed [59]	2009	n = 1284 CHW, n = 121 allopathic paraprofessionals, n = 19886 unqualified allopathic providers	Cross-sectional survey, population-based provider survey	Adequacy of drug provision	Multiple outcomes	Trained community health workers performed better than IPs
Bailey [99]	2002	n = 3518 women between 1990 and 1993	Training intervention for traditional birth attendants, quasi-experimental design, surveillance system of births, women interviewed postpartum by physicians	Recognition of maternal complications, referral rates	MCH	Mixed results (training+on rate, detection, and referral of postpartum complications; no evidence for overall increase in detection of complications, in referral to formal health care system, utilization of essential obstetric services)
Bang [61]	2005	n = 5919 live births in intervention villages	Retrospective analysis of intervention arm (39 villages) in home-based neonatal care trial in India	Knowledge and practice	Case fatality in LBW neonates	+ (mean of 19 indicators 80.5%)
Chalker [128]	2002	n = 22 matched pair intervention and control private pharmacies	RCT intervention pharmacies administered semi structured questionnaire pre and 4 months post intervention	Knowledge; change in practice for correct management of tracer conditions	STI, ARI, non-prescription requests for antibiotics and steroids	+
Chalker [88]	2005	n = 68 Hanoi, n = 78 Bangkok pharmacies, randomly selected	RCT intervention randomly selected pharmacies; five simulated client visits/pharmacy, assessed at baseline and month or more post-intervention. Three 3-month interventions sequentially with four months in between: 1. enforcement of regulations to emphasize prescription-only medicine legislation; 2. education; 3. Peer review (mandatory in Hanoi; voluntary in Bangkok)	Changes in practice for correct management of dispensing medication	Dispensing of steroids/antibiotics	Mixed results:+in Hanoi for reduction in dispensing illegal steroids, low dose antibiotics, sustained by means of peer review. Bangkok mixed results, only significant improvement was in reduction in illegal dispense of steroids
Chalker [86]	2000	n = 60 randomly selected private pharmacies in urban Hanoi.	Five simulated clients taught to adopt scenario that friend had urethral discharge; visited 60 randomly selected private pharmacies in urban Hanoi; semi-structured questionnaire to all people working in 60 pharmacies; questions asked, advice offered, treatment given were noted	Adequacy of advice, drug treatment, advising on partner notification, adequacy of STI treatment	STI	No adequate treatment given, poor partner notification, drug treatment
Chuc [90]	2001	n = 60 private pharmacies randomly selected	Cross-sectional survey assessing knowledge; practice assessed through simulated client method	Knowledge and practice	Childhood ARI	36% of cases handled dispensing of antibiotics according to guidelines; 41% used traditional herbal medicines; significant difference between knowledge/practice
Garcia [100]	2003	n = 14 districts randomly selected pharmacy workers	RCT intervention for training and support for management and prevention of STDs, standardized simulated patients visited clinics 1, 3, and 6 months after training	Recognition and management of STD syndromes	STD	+
Goldman [101]	2003	n = 64 trained and untrained midwives	Cross-sectional survey of midwives	Quality of care index	MCH	Trained and untrained midwives do not differ in quality of care index score
Greer [19]	2004	n = 245 outlets pre-interventions; n = 227 post-intervention	Intervention for training of PMVs, pre-post simulated patient design	PMV practices for simple or complicated malaria in children under five	malaria	+
Hamid-Salim	2006	n = 12525 village doctors trained in referrals and provide DOT	Community TB case detection data	Referrals to facility; treatment success rate	TB	11% of all TB cases with positive sputum referred by village doctors, 20–45% of patients on treatment during 1998–2003 received from village doctors, 90% treatment success rate
Jacobs [26]	2004	n = 405 men who sought treatment for urethral discharge at drug shops and private clinics	Cross-sectional survey	Quality of management of urethral discharge determined by: 1) treatment used in accordance with national guidelines; 2) number of properly managed patients (told to refer partner and use condoms or abstain from sex according to guidelines)	STD	Only 7% of clients were properly managed (28/405), and this was lower among clients seen at private clinics than at drug shops
Mignone [76]	2007	n = 503 allopaths; n = 421 non-allopaths; n = 74 registered medical practitioners (RMP)	Cross-sectional survey comparing three types of providers	Knowledge	HIV/AIDS	Allopaths had most knowledge, followed by non-allopathic providers and RMPs
Nsimba [35]	2007	n = 40 drug sellers in Tanzania	Training intervention took place one month after baseline data collected, 8-month follow up data	Knowledge, dispensing practices	Multiple outcomes	+
Oladepo [37]	2008	n = 110 PMVs and 113 households	Cross-sectional survey multi-stage random selection of respondents from 6 urban and 6 rural areas	Knowledge of government policy on malaria drugs; 2 knowledge change in policy concerning cloroquine or ACTs	malaria	1. 43.1% were aware of new government policy on AMDs; 2. 24.5% knowledge of change in policy concerning chloroquine or ACTs
Peltzer [41]	2006	n = 233 traditional healers in four communities in South Africa	Intervention for training in HIV/AIDS, STI, and TB prevention over 3.5 days, as well as supervisory follow-up visit, pre-post study	1. Knowledge, 2. HIV and STI management strategies, conducting risk behavior assessments, counseling, condom distribution, community HIV/AIDS and STI education, record keeping	HIV/AIDS, STI, TB	+
Poudyal [78]	2003	n = 48 trained traditional healers, n = 30 untrained traditional healers	Cross-sectional intervention design comparing trained and untrained traditional healers, received training in western medicine	1. Knowledge about preventive measures for various illnesses 2. Knowledge about signs and symptoms of various illnesses	Malnutrition, ARI, Diarrhea, Night blindness, HIV/AIDS	+
Rowen [81]	2009	n = 45 traditional birth attendants	Training intervention, semi-structured surveys conducted pre/post intervention.	Knowledge and practice	MCH	+
Stenson [94]	2001	n = 214 pharmacists	Intervention pre−/post study, intervention included inspections of pharmacies, information, and distribution of documents to drug sellers	1. Availability of essential materials, 2. Information to customers, 3. Packaging of drugs	Multiple outcomes	+ (information to customers increased from 35% to 51%, mixing of drugs in same package went down from 17% to 9%)
Syhakhang [98]	2004	n = 59 drug sellers and n = 278 exit clients	Mixed-methods cross-sectional study	1. Definition of drug quality, 2. Drug practices	Multiple outcomes	Inadequate scientific drug knowledge, only 1 drug seller knew definition of drug quality, 2 knew correct temperature for drug storage, 44% knowledge on drug labeling, 73% could read expiration date, 58% bought drugs from unauthorized source, 73% did not worry about quality of drugs
Syhakhang [97]	2004	n = 115 private pharmacists	Cross-sectional survey of pharmacists in 1997 and 1999	Drug quality according to standards of British and US pharmacopoeias	Multiple outcomes	Substandard drugs decreased from 46% to 22% between 1997 to 1999.
Tavrow [48]	2003	n = 101 informed outlets, n = 151 control outlets	Intervention training wholesalers, evaluated using mystery shoppers posing as caretakers of sick children at 252 drug outlets	Practices	Malaria	+32% visiting informed outlets sold first-line drug for malaria compared to 5% at control sites
Tawfik [50]	2006	n = 386 traditional birth attendants, 321 drug shops, 281 traditional healers, 74 private clinics, 19 maternity homes, 17 ordinary shops	Intervention pre-post test with simulated client visits	Practices	Child outcomes	+
Taylor [51]	2001	n = 581 samples of 27 different drugs from 35 pharmacies in Nigeria	Cross-sectional random collection of drug samples	Drug quality assessed according to pharmacopoeia requirements	Multiple outcomes	48% of samples did not comply with set pharmacopeia limits
Viberg [55]	2009	n = 94 drug sellers	Cross-sectional study of drug sellers, face-to-face interviews and simulated client method to assess practice	Practices	STI	Medications dispensed in 78% of male and 63% of female simulated client visits, dispensed drugs that were recommended in Tanzanian guidelines for syndromic management of urethral or vaginal discharge in 80% of male and 90% of female cases, dosage regimens incorrect and complete syndromic management rarely provided
Wolfe [103]	2005	n = 30 pharmacies attended training	Pre/post youth friendly training intervention with pharmacists/clerks, mystery client visits rated pharmacists, compared trained vs. untrained pharmacists	Providers trusted, friendly, practices, counseling	Youth health	+

Notes: Under significance,+only indicated for intervention studies to indicate positively impact quality; STI = sexually transmitted infection; ARI = acute respiratory infection; PMV = patent medical vendors.

To assess the quality of IPs, we included studies that reported on technical or perceived dimensions of quality. Technical quality included adequacy of health provision determined by existing guidelines (i.e. national guidelines), counseling, referral rates, and health practice. Studies that reported on perceived quality, as reported by clients, were also included in the literature review. We excluded studies that only reported on provider-specific characteristics, such as only knowledge, but reported knowledge level when combined with other quality predictors. Descriptive summary statistics such as percentage of utilization of IP sector across countries was used to assess size of informal providers sector. Descriptive statistics such as treatment rates, success rates, referral rates, and difference in means was used to assess quality of informal providers (see Table 3).

## Results

A total of 334 references were gathered from the past eleven years (2000–2011). Upon reviewing abstracts, 103 were found to be pre-2000 and were therefore excluded from further analysis. In order to ensure the literature review reflects the current state of informal providers, a 2000-publishing cut-off was chosen; however, studies that used data pre-2000 were used for contextual and background information. Two hundred and thirty-one studies were included in the review: 122 were chosen for data abstraction. The remaining 109 references were indirectly relevant to the topic and provided additional context and background for the review. Regionally, almost half of the studies (48.0%) of studies were from sub-Saharan Africa [3]–[56], followed by 23.5% in South Asia [57]–[83], 12.6% in Southeast Asia regions [84]–[98], and 4.2% in Latin America [99]–[103]. Additionally, almost 12% of articles covered multiple regions [104]–[115]. A total of 26, all low-or middle-income (LMIC) countries were represented in the references included for abstraction.

### The Scope of Practice of Informal Providers

An IP’s scope of practice is altered according to contextual variation. The robustness of the regulatory framework, strength of enforcement mechanisms, influence of cultural traditions, condition of the formal health infrastructure, and the demand for services all shape IPs’ activities and may vary on a national or regional level. Informal providers operate in a variety of health areas. The literature search most commonly identified studies with “multiple disease areas” (n = 41/118). In disease-specific studies, maternal and child health was the most common area of study with 25 papers, followed by malaria (n = 22), HIV/AIDS (n = 10), and reproductive health (n = 11). Tuberculosis, mental health, and asthma treatment all had 5 or less studies dedicated to the outcome and IPs.

Across the studies, drug sellers (n = 45) were the most common type of IP, followed by papers that discussed multiple kinds of IPs (n = 30), TBAs (n = 18), village doctors (n = 19) and finally those that studied an informal brand of CHW (n = 5). Depending on the regulatory and enforcement environment, many drug sellers operate beyond their legal capacity by selling prescription-only medications as well as offering diagnostic and therapeutic medical advice [88]. Regulatory infringements are commonplace among drug sellers [15]. Some drug sellers have itinerant or mobile vending operations, but the extent of this practice is unclear and likely due in part to research challenges to completely capture the process [15].

The activity of village doctors and traditional medical practitioners is less consistent. Some village doctors have practices nearly indistinguishable from those of licensed allopathic physicians [116]. In some contexts, traditional practitioners primarily utilize non-allopathic modalities such as massage and herbal medicines [96], while in other situations they might supplant allopathic care completely [117]. An urban-rural division is apparent in some studies, which demonstrate that more rural populations are more likely to use village doctors and traditional practitioners than their urban counterparts [25], [38], [68].

Traditional birth attendants (TBAs) play a role in pre- and post- natal care that ranges from acting as the sole caregiver to supporting trained birth attendants [112]. The term TBA encompasses providers of varying skill and training. In some cases a TBA is simply a family member while in other cases she practices regularly and relies on the work for an income, representing a spectrum of practice so broad that some researchers contend the term “TBA” is inappropriate to describe such heterogeneous pool [77]. The effectiveness of training programs for TBAs is the subject of an ongoing debate [113].

IPs generally practice poor preventive medicine, particularly IPs whose practice primarily consists of dispensing products or services in discrete units (e.g., drug sellers) [59], [69], [118]. Even those IPs who offer a more continuous and preventive brand of medicine (e.g., village doctors) might have a limited practice, centered around emergent situations, due to a general inability to pay for long-term health care in their community [119], [120].

### Size and Utilization Patterns in the Informal Health Care Sector

Studies that discuss the size of the IP sector attempt to understand what proportion of all providers in a given geographical region are informal. Utilization patterns examined care-seeking behavior and quantify patients’ preferences for IPs. Relatively few studies (n = 24) document the utilization of IPs for health care services, and even fewer (n = 5) describe the size of the IP sector in relation to the health care provider pool overall. This review looked at studies in both categories, and is presented in Table 2.

Five studies reported the portion of all providers in a nation or region that are informal, ranging from 51% to 96%. The methodologies in these studies have inherent limitations including cross-sectional designs, small sample size, and unclear classifications of informal providers, which may explain the wide variation. In Bangladesh, researchers estimated that 87% of providers were informal [59], while in the rural region of Chakaria, 96% of all providers were informal [64]. In India the informal sector was found to be between 51–55% of all providers [68], [80]. In Uganda, 77% of providers were found to be informal [32].

Utilization was defined in most cases as the first choice for care, but in other studies utilization was defined as the exclusive choice or as having played any role in care. Utilization estimates from 24 studies in the literature of IP for healthcare services ranged from 9% to 90%, depending on the country, the disease in question, and methods of measurement (see Table 2). For example, in Bangladesh, utilization of IPs was consistently high across studies ranging from 60%–77%. Using a large nationally representative survey of Bangladesh, one study found that 65% of individuals went to an informal healthcare provider for care [75]. Studies of Kenya, on the other hand, ranged from 9%–33% for IP consultation for fevers [6], [20].

Within countries, some studies have consistent findings (i.e. as in Nigeria, where four studies found results within ten percentage points of one another) [9], [121] while others arrive at vastly different results: three studies from Uganda featured a spread of over 50 percentage points [9], [26], [32], [37], [121], [122]. In some cases, utilization data seemed to reflect the varying popularity of different types of IP, with studies including drug sellers consistently demonstrating higher utilization numbers (see Table 2).

### Quality of Informal Providers

Out of the 122 articles, we identified 29 that reported on a clinical quality outcome (see Table 3). Most studies on quality are abstracted from interventions (16/29 articles), and 13 articles were from cross-sectional studies. Seventeen of the articles had a comparison group in order to assess quality of providers, five of which included a formal sector control. In the case of intervention studies, almost all reported on baseline characteristics and post-intervention results (pre-post design), or compared experimental vs. control groups.

While measuring quality of care is difficult, there are a variety of methods used across the studies. Eight studies used mystery clients, or simulated standardized patients, to assess quality [86], [88]. The standardized patient methodology is considered to be the “gold standard” for measuring clinical quality for a number of reasons: First, other methods are subject to observational biases (i.e. direct clinical observations), recall bias (patient exit interviews), and informational bias (i.e. chart abstraction). Standardized, simulated patients are able to give a more complete and valid comparison across a wide range of providers. We identified studies that reported technical or perceived dimensions of quality (as opposed to reporting on provider-specific characteristics, such as reporting only on provider knowledge). Technical quality included adequacy of health provision determined by existing guidelines (i.e. national guidelines), counseling, referral rates, and health practice. Studies that reported on perceived quality, as reported by clients, were also included in the literature review. Quality predictors identified in the studies include: 1) provider knowledge and skills; 2) adherence to clinical guidelines; 3) patient satisfaction.

Provider knowledge was variable across studies. Compared to the formal sector, training among IPs are limited across multiple health outcomes, and they lack necessary training and capacity to provide basic curative services [59], [76], [86]; however, two studies also found no difference in clinical quality when comparing formal and informal providers [26], [101]. Compared to community health workers and allopathic paraprofessionals, IPs scored lower on adequate drug provision based on provider surveys [59]. In addition, there were also gaps in knowledge and practice, perhaps due to lack of resources, access, and drug availability [19].

Studies also found poor adherence to clinical national guidelines [26], [51], [55], [90]. For example, in Vietnam, Chuc (2001) [90] found that only 36% of pharmacists dispensed antibiotics according to national guidelines; 41% used traditional medicines. Jacobs (2004) [26] found that only 7% of clients were properly managed according to national guidelines, including both clients who went to private clinics as well as drug shops.

Interventions assessed the impact of training on the clinical quality of providers. Fourteen out of sixteen training interventions resulted in positive quality outcomes (either pre/post test or increased quality compared to comparison groups) (see Table 3). Two studies reported mixed results for interventions [99]. Chalker [88] found that enforcement of regulations, increased education, and peer review effectively changed practices for management of dispensing medication in Hanoi; however, in Bangkok, the intervention only improved reduction in illegal dispensing of steroids, while other practices remained the same pre and post-intervention. The authors attributed this to the voluntary nature of peer review in Bangkok, as opposed to mandatory activities in Vietnam, suggesting the peer review process as an important component of sustained change [88]. Based on these studies, training improved quality, but longer follow-up periods are necessary in order to address issues of sustainability. One study on a youth-friendly training intervention that was implemented in 30 pharmacies reported on perceived patient satisfaction and relationship with providers [103]. Mystery clients (blinded to who received and did not receive intervention) visited trained and untrained pharmacists and rated them on perceived trust, friendliness, practices, and counseling. Pharmacists who received youth-friendly training were rated higher across all four domains of patient satisfaction.

### Reasons People Use Informal Providers: Convenience, Affordability, and Culture

There are three principal reasons for using IPs: convenience, affordability, and social and cultural effects. Compared to either the public or formal private sectors, IPs have flexible working hours and are likely to be open at all hours, more likely to have medicines in stock, generally geographically closer, and offer more rapid service. A study in West Bengal noted that the two most commonly cited reasons for visiting an IP were the proximity of location and the longer and more flexible business hours (74% and 65% of respondents, respectively) [123]. In a review of drug sellers, Goodman et al. (2007) [16] reported that IPs were closer, faster, and better stocked than formal caregivers [15], [16]. IPs are typically considered a one-stop shop, and men seeking treatment for STDs in Thailand cited a preference for IPs due to their convenience [124].

IPs are more affordable than private sector providers and may even be more affordable than publicly administered free clinics when transport and informal payments are considered [6]. A study in Bangladesh found that 61% of respondents cited affordability as an important reason for visiting an IP [123]. One way IPs lower prices is to repackage medications into smaller, more affordable units [15]. While formal providers may offer subsidized or free goods and services, researchers report that irregularity in such benefits reduces their overall attraction [30]. While formal providers charge for diagnostics, procedures, and fees, IPs primarily accrue payment through up-front consultation fees and the dispensing of medications [63]. This matters to patients: one study found that 73% of women who visited drug sellers were not worried about the quality of the drug because cost was more important [98].

IPs may also accept in-kind payment when patients have no cash. Many studies cite transport costs as an indirect but important element that increases the cost of care by formal providers, who are often located farther from the patients’ homes [71]. Indirect transport costs may exceed direct costs of care associated with formal providers [6], [64], [125].

A study in Bangladesh found that the median cost of treatment of visiting a formal physician was five and fifteen times higher compared to visiting a village doctor or traditional healer, respectively [64]. The study did not control for varying levels of sickness; therefore, it is possible that patients self-select into formal providers when they are sicker, thereby incurring higher treatment costs on average in the formal sector. However, other evidence from Bangladesh suggests that even when illnesses are serious enough to require multiple visits, IPs remain substantially cheaper than their formal counterparts [63].

In addition, IPs often possess social and cultural advantages over formal private and public providers due to their status in the community. For example, they are subject to a greater degree of perceived accountability due to their geographic and social proximity to patients; they can better evaluate the trustworthiness of individuals and may offer creative financing for goods or services as a result; and their experiences, qualifications, and track record are all noted within a community, resulting in trust and respect [126]. The scope of practice of IPs is uniquely tied to community-level factors, more so than for formal providers [2]. IPs run smaller, more localized operations [49]. Much of their practice is contingent on the maintenance of good relationships with their communities. This aspect of practice for IPs is tied to their more forgiving and creative payment policies, whereby patients might be put on a sliding scale or deferred payment plan in accordance with their ability to pay. Such practices tend to buttress their role in the community, and thereby strengthen their business position [56].

Researchers have noted that IPs could be perceived as a more suitable option by family decision-makers, particularly for women’s reproductive health care [30]. IPs that perform home visits and are well known within the community offer a degree of security that is difficult for more centralized formal providers to achieve when dealing with sensitive health issues [126]. While public clinics may see significant staff turnover and staff may be relatively young and inexperienced, IPs offer a more trustworthy and stable environment, where staff are part of the community and reputations are well established [30].

### Recommendations for Informal Providers

We evaluated the recommendations in each study regarding IPs, and created a classification system for recommendations that was informed by previous work on the topic [16]. The categories include: 1) *education interventions* including capacity building exercises and training programs; 2) *oversight interventions* including regulation, enforcement, and registration; 3) *process intervention*s including collaboration and engagement; 4) *recognizing the social and cultural value* that IPs offer foremost; 5) *conducting further research* on IPs (no other recommendations provided); and 6) reducing the need for IPs by *improving access to formal providers.*

Using this categorization, we abstracted the most important two recommendations per reference and recorded these in a database. Next, we tallied the overall frequency of recommendations in an attempt to characterize the literature’s view on IPs. Of 195 papers that had recommendations, the most common (66 studies) were *educational interventions*, including capacity-building training programs for IPs, *patient education programs*, and *continuing education requirements*. Sixty-one publications called for *oversight interventions* including increased government oversight and regulation, as well as *professional associations* and other means for quality assurance. *Process interventions* were the third most-recommended intervention type (38 studies). These include building dialogue and understanding, and fostering working relationships between formal and informal providers. Eleven papers suggested that governments and researchers should recognize the social or cultural value of IPs, and ten papers only called for more research on IPs. Improving access to formal providers was the least cited recommendation (nine studies).

## Discussion

In the developing world, informal providers represent a significant portion of the healthcare system. This review is the first comprehensive view of studies focusing on the size, scope, and quality of IPs, and is timely given this growing sector of the health system. This review revealed that studies on informal providers are limited in their methodology, scope, and descriptive ability, and evidence regarding the size, utilization, and quality of the informal sector is scarce. While there is wide variation across studies and countries on how large the sector truly is, it is clear that informal providers make up a large proportion of the health care provided, particularly among poor populations, and they fill a significant role in the health market. IPs covered a broad range of health areas including maternal and child health, malaria, HIV/AIDS, and reproductive health. Informal providers are serving multiple functions in the delivery of healthcare and include drug sellers, traditional birth attendants, and village doctors.

There was a dearth of information on the size of the informal provider sector as well as utilization rates. This study found huge variations in the size of the informal provider sector (51% to 96%), and an even greater range in utilization patterns (9% to 90%). These significant ranges may be explained by study variations and regional differences. Variations in sample size and sampling techniques make it difficult to compare across studies. While some studies were nationally representative, the majority of studies were small in sample and regionally specific. For example, a focused-study in a poor region of a country known to have a high proportion of informal providers would provide much different results compared to a national survey of providers given that we know that the poor are more reliant on this sector. Types of informal providers in the study also differed across region; some studies were more inclusive and surveyed a number of types of the formal sector (i.e. village doctors, medical vendors, compounders), while others focused specifically on one type of provider. This makes it difficult to truly assess the proportion of informal providers in a given health market. Moreover, in order to fully summarize the size of the informal provider sector, a deeper understanding of local health markets and the role of the formal sector is critical. This remains a significant challenge, and is an opportunity for future health systems research.

In addition, this study highlights why people choose to go to informal providers. Poor populations are most likely to seek care from this sector [1], and convenience, affordability, and cultural factors were the most commonly cited reasons. Informal providers fill a significant gap in healthcare delivery particularly for individuals without the means to travel to public facilities or afford health insurance. Travel costs are an indirect, but important element, in increasing the cost of care from the formal sector. In countries such as Bangladesh, public facilities are not well stocked with appropriate drugs and are typically overwhelmed with long lines and a scarcity of physicians and nurses. In particular, while affordability and convenience may not seem surprising in the decision to visit an informal provider, as documented by the literature, the role of culture and values plays a particularly significant role. Cultural values dictate patient preferences, and the status and reputation of providers in communities, trust in informal providers (and distrust of the formal sector), and increased anonymity given certain visits (i.e. family planning) are all factors that influence where people go for medical care. Governments, donors, and NGOs should take these factors in to account when developing interventions that target the poor.

Quality was variable across contexts, and studies demonstrated that there was generally low provider knowledge, inadequate drug provision, and gaps in knowledge and practice. One study found that even with adequate training, clinical quality trails behind knowledge due to lack of resources, access, and drug availability [19]; in other words, provider knowledge is insufficient as a measure of clinical quality. Moreover, drug vendors are the most commonly cited informal provider, despite findings that the majority lacked training to provide basic primary care services [59]. Engaging these providers in to the mainstream health system to provide appropriate drug dosages and referrals may be a feasible way to broadly reach poorer populations.

This review also highlighted recommendations to better support informal providers. Educational interventions, including capacity building exercises and training programs, were the most commonly cited strategy for engaging IPs. Other recommendations included increased government oversight and regulation, process interventions, and finally, greater access to formal providers. Given that affordability and convenience were reasons given for tapping in to IPs, the formal sector may incorporate strategies for addressing these gaps to better reach the poor. A systematic review of the literature identified the most effective interventions as ones that combined educational training with improving market conditions, such as provider incentives and accountability [127]. Interventions that do not rely solely on individual-level behavior changes, but also combine market-based approaches and rigorous evaluations, may prove to be most successful in improving quality. In addition, collaborating with a local advisory board as well as partners who were committed to long-term change were important lessons learned from implementing a successful intervention [103].

There are a number of limitations in this review given the paucity of research on informal providers. This review aimed to be inclusive of studies on informal providers, and therefore, a range of study designs and samples are used. Comparability across studies was difficult. First, cross-sectional studies assessing interventions is limited in its’ ability to establish causality. Studies lacking baseline data or comparison groups, including pre/post data or IPs vs. non-IPs, are unable to truly assess the effects of interventions. Second, because some studies in the review include limited sample size, external validity and generalizability may be an issue. Study results and implications should not be extrapolated outside the study sample. In addition, there are a number of potential limitations in the review. For example, because of publication bias, the review may overestimate specific interventions- statistically significant interventions may be more likely to be accepted for publication. However, this review explores both peer-reviewed and grey literature, and therefore may have been more likely to capture non-significant studies compared to only searching published literature. In addition, because of the wide variation in study designs and types of providers included in the literature review, the heterogeneity of the sector is not explored in this review. This warrants further investigation into subsets of informal providers and analysis of the types of quality issues specific to providers.

Limitations in the literature suggest that further research is needed in order to fully understand this growing sector. In particular, because informal providers typically work outside the purviews of a regulatory framework, there is little information on the quality of services delivered by IPs. What information there is suggests that quality of care is poor, and this may pose risk to the most vulnerable populations. Specific research studies should focus on better understanding local health markets as well as documenting key characteristics of the informal provider sector. This research is needed in a number of areas:

First, the definition of informal providers needs further assessment. Because the formal sector’s increasing recognition that IPs play a significant role, engagement with this sector has started to change and therefore definitions need to be adapted accordingly. While the definition used in the review may be debatable, it was needed to delineate which studies and types of providers should be included under the term ‘informal provider’. Further public discussion on this is needed. Second, future studies should focus on specific types of informal providers, and specific services, in order to help with strategies of engagement. The informal provider sector is a heterogeneous group, and targeted interventions and policies can reflect this. Third, understanding user choices and patient knowledge is important. Studies would benefit from understanding networks of providers and patients and what are the decision-making factors that influence choice in care. Fourth, future studies need to focus on quality of providers, using robust study designs and measures. This literature review suggests that knowledge, for example, is not an adequate measure of quality and needs to be combined with measures of technical competence and perceived quality of care. Lastly, a deeper understanding of local health markets, the relationship with the formal sector, and a mapping of providers in a community will help researchers, donors, and policy-makers target care appropriately. While many questions remain, it is clear that greater attention is needed to engage informal providers in order to assure that patients in developing countries, particularly poor patients, have access to appropriate and safe medical care.
