# Correction: What Is the Role of Informal Healthcare Providers in Developing Countries? A Systematic Review

**DOI:** 10.1371/annotation/93bc7847-5c4e-4b66-8923-500aa4fa4da5

**Published:** 2013-09-13

**Authors:** May Sudhinaraset, Matthew Ingram, Heather Kinlaw Lofthouse, Dominic Montagu

A funding organization was incorrectly given in the Funding Statement. The Funding Statement should read: "Results for Development's Center for Health Market Innovations provided funds to complete this systematic review. The funders had no role in study design, data collection and analysis, decision to publish, or preparation of the manuscript." 

